# Niche–dependent sponge hologenome expression profiles and the host-microbes interplay: a case of the hawaiian demosponge *Mycale Grandis*

**DOI:** 10.1186/s40793-024-00563-8

**Published:** 2024-04-08

**Authors:** Fang Liu, Taewoo Ryu, Timothy Ravasi, Xin Wang, Guangyi Wang, Zhiyong Li

**Affiliations:** 1 State Key Laboratory of Microbial Metabolism, School of Life Sciences & Biotechnology, Shanghai Jiao Tong University, 200240 Shanghai, P. R. China; 2 Joint International Research Laboratory of Metabolic & Developmental Sciences, Shanghai Jiao Tong University, 200240 Shanghai, P. R. China; 3 Marine Climate Change Unit, Okinawa Institute of Science and Technology Graduate University (OIST), 1919-1 Tancha, Onna-son, 904-0495 Okinawa, Japan; 4 Department of Microbiology and Cell Science, Institute of Food and Agricultural Sciences, University of Florida, 32611 Gainesville, FL USA; 5 School of Environmental Science and Engineering, Tianjin University, 300072 Tianjin, P. R. China

**Keywords:** *Mycale Grandis*, Hologenome, Gene expression, Host-microbe interplay, (Meta)transcriptome, GeoChip microarray

## Abstract

**Background:**

Most researches on sponge holobionts focus primarily on symbiotic microbes, yet data at the level of the sponge hologenome are still relatively scarce. Understanding of the sponge host and its microbial gene expression profiles and the host-microbes interplay in different niches represents a key aspect of sponge hologenome. Using the Hawaiian demosponge *Mycale grandis* in different niches as a model, i.e. on rocks, on the surface of coral *Porites compressa*, under alga *Gracilaria salicornia*, we compared the bacterial and fungal community structure, functional gene diversity, expression pattern and the host transcriptome by integrating open-format (deep sequencing) and closed-format (GeoChip microarray) high-throughput techniques.

**Results:**

Little inter-niche variation in bacterial and fungal phylogenetic diversity was detected for *M. grandis* in different niches, but a clear niche-dependent variability in the functional gene diversity and expression pattern of *M. grandis* host and its symbiotic microbiota was uncovered by GeoChip microarray and transcriptome analyses. Particularly, sponge host genes related to innate immunity and microbial recognition showed a strong correlation with the microbial symbionts’ functional gene diversity and transcriptional richness in different niches. The cross-niche variability with respect to the symbiont functional gene diversity and the transcriptional richness of *M. grandis* holobiont putatively reflects the interplay of niche-specific selective pressure and the symbiont functional diversity.

**Conclusions:**

Niche–dependent gene expression profiles of *M. grandis* hologenome and the host-microbes interplay were suggested though little inter-niche variation in bacterial and fungal diversity was detected, particularly the sponge innate immunity was found to be closely related to the symbiotic microbes. Altogether, these findings provide novel insights into the black box of one sponge holobiont in different niches at the hologenome level.

**Supplementary Information:**

The online version contains supplementary material available at 10.1186/s40793-024-00563-8.

## Background

Sponges (Phylum Porifera) are benthic, sessile metazoans across the world’s oceans and freshwater environments, encompassing more than 8,500 formally described species [1, 2]. Genomic evidence places sponges at the base of the metazoan phylogenetic tree, supported by fossil records dating back 580 million years [1]. One sponge host and its symbiotic microbiota form a sponge holobiont [2, 3]. Their collective genomes, including both the sponge and its microbiome, are known as ‘sponge hologenome’ [4]. Recent research by Vargas et al. provided the holistic evidence for the sponge body-plan reorganization in response to microbiome perturbations, underlining the interaction between the sponge and its microbiota [5]. This intricate relationship between sponge hosts and their symbiotic microbes renders sponge holobionts as exceptional models for studying animal-microbe symbioses [6]. Beyond their scientific interest, sponge holobionts serve various functions in marine ecosystems and biotechnology e.g. geochemical cycling [4], production of novel marine enzymes and natural products with potential applications [7, 8].

The global sponge microbiome project underscores the vital role of sponge holobionts as reservoirs of exceptional microbial diversity, contributing significantly to the world’s oceanic microbial diversity [3]. This project also indicates that sponge host phylogeny predominantly influences the complexity of its symbiont community though. geographic or environmental factors show impacts on sponge microbial community composition [9–11]. Core sponge microbiomes, characterized by generalist symbionts exhibiting amensalistic or commensalistic interactions, demonstrate notable stability against environmental or geological factors [3]. Functional equivalence and evolutionary convergence among complex sponge microbial symbionts have also been proposed [12], underlining their essential roles in the sponge holobionts. The functions of sponge holobionts pivot on the stability of host-symbiont interactions and their response to environmental changes. Although Erwin et al. [13] have highlighted the temporal stability of complex host-microbe symbioses in a temperate, seasonal environment, the present researches primarily focuse on symbiotic microbes within sponge holobionts, with limited exploration of the sponge host changes, especially at the hologenome level [2, 3, 11–13]. This indicates a significant knowledge gap in understanding sponge host gene expression and the host-microbe interplay across diverse ecological niches.

The marine sponge *Mycale grandis* (formerly *Mycale armata*) is widely distributed in highly diversified environments (here described as different niches) around Hawaii as an encrusting sponge, such as on rocks, on the surface of reef-building corals such as *Porites compressa* [14], or under the mats of macroalga *Gracilaria salicornia*. Our previous investigations (including the sampling sites selected in this study) illustrated that *M. grandis* had relatively consistent bacterial and fungal communities [15, 16]. Therefore *M. grandis* is an ideal model for investigating the sponge holobiont’s relationship with niches. It can be hypothesized that the sponge hologenome’s expression and host-microbes interplay of a sponge holobiont are niche-dependent, even though the symbiotic microbial community remain relative stable across different niches. In order to provide evidence for this hypothesis, the microbial community structure, functional gene diversity, expression pattern and the host transcriptome of *M. grandis* in different niches were compared in this study using an integrated approach including open (pyrosequencing, (meta) transcriptomics) and closed (GeoChip microarray) platforms of high throughput technologies. This study unravels the intricate interplay between a sponge holobiont and its environmental niches by integrating data on the host’s gene expression with the profiles of its associated bacterial and fungal communities.

## Materials and methods

### Sponge sampling and nucleotide acid preparation

**Fig. 1:**
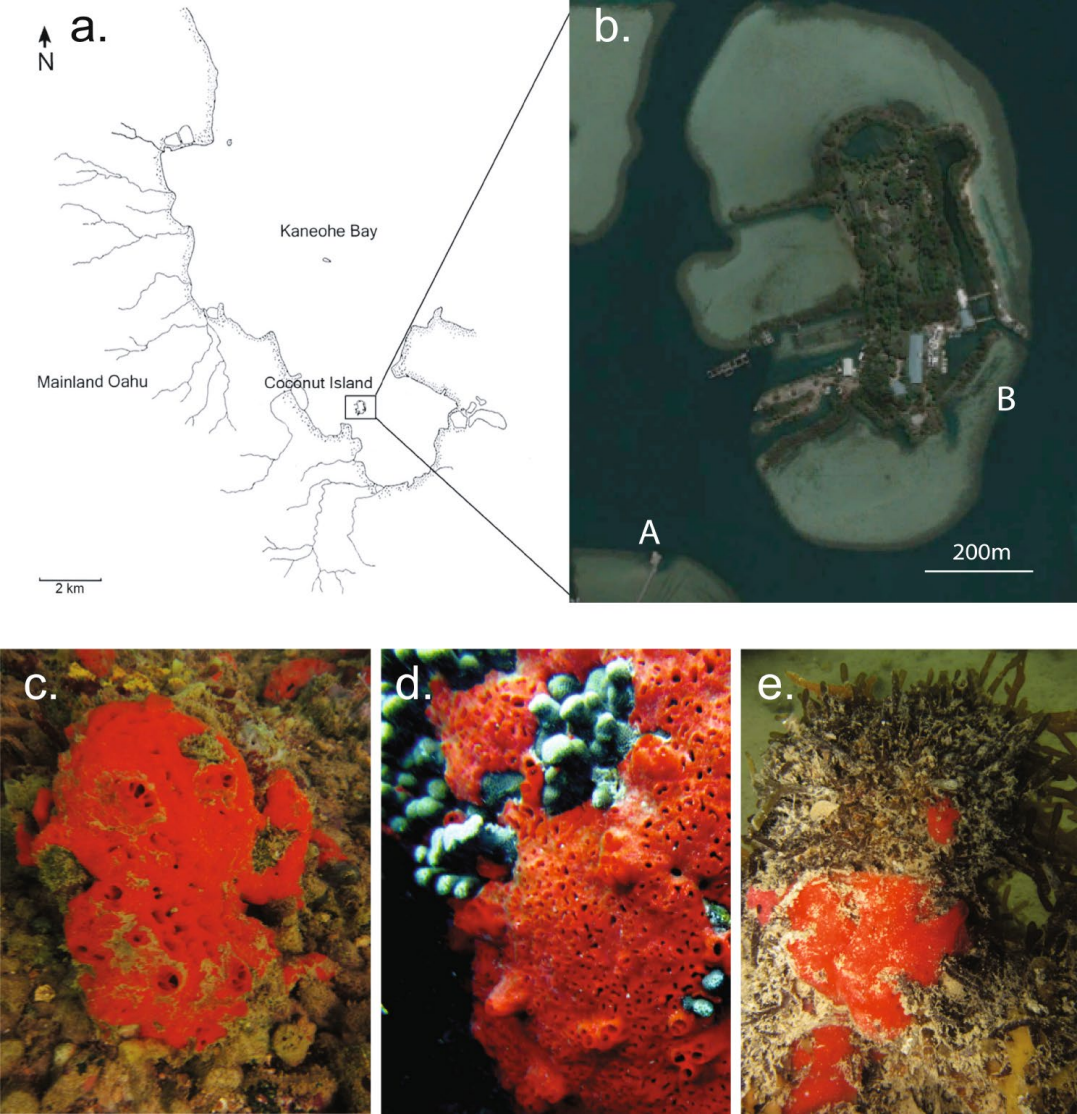
Sampling map. **(a)** Location of Coconut Island, **(b)** Aerial image sourced from Google Earth. Spot A, 21°25’47”N, 157°47’31”W. Spot B, 21°25’57”N, 157°47’12”W. **(c)*** M. grandis* on rocks (spot A), **(d)*** M. grandis* on the surface of coral *P. compressa* (spot B), **(e)*** M. grandis* under alga *G. salicornia* (spot B, inverted view)

Sponge *Mycale grandis* samples in three different niches, i.e. on rocks, on the surface of coral *Porites compressa* and under alga *Gracilaria salicornia*, were collected around Coconut Island, Kaneohe Bay, Oahu, Hawaii, in May 2013 (Fig. 1). Sponge specimens were collected from 3 different individuals from each niche, respectively, at a depth of 0.5–1.5 m and put in sterile zip bags underwater. Once on board, the sponge specimens were immediately rinsed with autoclaved artificial seawater. After cutting into pieces thinner than 5 mm by clean scalpels, the sponge samples were put into RNAlater (Qiagen, Germany), and then stored at -80 °C. Coral branches (*P. compressa*) and algal tissues (*G. salicornia*) were collected in triplicate along with the sponge sampling and kept in RNAlater using the same procedure as sponge samples. The sampling permission of sponges, corals and algae was issued by Hawaii Division of Aquatic Resources with the tracking number SAP-2012-71. Ambient seawater ($\sim$ 5 L) was collected simultaneously and filtered by 10 μm and 0.22 μm filters (Millipore, USA) subsequently. The filters were then preserved in falcon tubes with 15mL RNAlater, and then kept at -80 °C.

For rRNA-based sequencing and GeoChip analyses, the nucleic acid samples were prepared as follows: all biological tissues were homogenized by a FastPrep 24 machine (MP Biomedicals, USA) following manufacturer’s instructions to facilitate the lysis process. Total DNA and RNA were extracted using AllPrep DNA/RNA Mini Kit (Qiagen, Germany). The absence of DNA contamination in RNA samples was confirmed by performing 16 S rRNA gene PCR [17] and 18 S rRNA gene PCR [18]. The total RNA samples of *M. grandis* and seawater were converted into ds cDNA with random hexamer primers using RevertAid Premium Double Stranded cDNA Synthesis Kit (Thermo Scientific, USA). DNA samples of different coral and algal individuals (*n* = 3) were pooled together because they were used only as a simple control for sponge microbiome analysis. To enable sponge transcriptome sequencing, the total mRNA of sponge was extracted and purified per the method described by Riesgo et al. [19] was used for the sponge samples’ mRNA purification.

### 454 pyrosequencing and data analysis

The bacterial V3-V4 16 S rRNA fragments and fungal D1-D2 28 S rRNA fragments were amplified from sponge cDNA samples (biological triplicates for each niche) (Table S1) [17, 20]. In addition, DNA of seawater was used to serve as a control/background for sponge datasets. Sequencing on Roche GS FLX + was carried out in Personalbio Inc. (Shanghai, China). The forward amplicons were kept for further analysis. For 16 S rRNA sequencing data, the reads were trimmed to 300 nt and a cutoff of the expect error > 0.5 was used to filter out the low-quality reads. USEARCH ‘derep_fulllength’ command with the ‘-sizeout’ option was used for dereplication. Quantitative insights into microbial ecology (QIIME) pipeline v1.9 was then used for bacterial OTU picking and diversity analysis following the official tutorial [21, 22]. A cutoff of 3% dissimilarity was used in open-reference operational taxonomic units (OTU) picking (pick_open_reference_otus.py). Oligotyping program was leveraged to dissect the predominant bacterial members on single-nucleotide level [23]. For fungal 28 S rRNA sequencing data, the reads were trimmed to 250 nt and a cutoff of the expect error > 0.5 was used to filter out the low-quality reads. For fungal community profiling, UPARSE pipeline (usearch 8.1) was used for further quality control and OTU picking by a 99% similarity cutoff [24]. The taxonomic annotation of representative sequences of bacterial OTUs and fungal OTUs was carried out using ribosomal database project (RDP) Classifier with default settings. The 16 S rRNA reference version 10 and Fungal LSU training set 11 were used in RDP analysis, respectively [25]. The biological observation matrix (BIOM)-format OTU table was imported into QIIME 1.9 for statistical analysis. Due to the incompatibility between the default aligning algorithm of QIIME and the 28 S rRNA sequences during UniFrac analysis, the alignment of fungal sequences was analyzed with MAFFT algorithm in QIIME. The predominant OTUs were also deconstructed by Oligotyping.

### GeoChip microarray analysis

The total DNA and ds cDNA of sponge specimens (*n* = 2 × 3 × 3, biological triplicates for each niche), along with the total DNA of coral and algal samples, were amplified and quantified using GeoChip 4.6. Hybridizations was performed at 42 °C with 40% formamide for 16 h on a MAUI hybridization station (BioMicro, USA). Signal conversion, raw data collection and data normalization were provided by GLOMICS (https://www.glomics.com/gch-tech.html). In this study, spots with both DNA and cDNA signals were considered as positive and used for further analysis. The initial data analysis was carried out with the pipeline for GeoChip named Microarray Data Manager (GeoChip IV) (http://ieg2.ou.edu/NimbleGen/). The ordination analysis, univariate and multivariate statistics were performed using Past 4.02 [26] and R-3.3.3 [27], as well as heatmap visualization.

Our goal was to investigate the activity of functional genes, i.e. transcriptional richness, therefore to ensure accuracy, we used the ratio of cDNA to DNA to rule out the overestimation of transcripts due to a high copy number of genes. The expression ratio was determined by the ratio of cDNA to DNA signals for each spot. This was done to enable a comparison of the expression levels of different variants. A variant was classified as a high-expression-ratio variant if its expression ratio exceeded the median expression ratio of the sample group.

### Establishing sponge host’s transcriptome based on RNA-Seq

*Mycale grandis* mRNA libraries (n = 3 × 3, biological triplicates for each niche) were prepared using TruSeq RNA Sample Prep Kit (Illumina, USA) and sequenced using HiSeq2000 technology (Illumina, USA) at GENEWIZ company (Suzhou, China). Before *de novo* assembling, low quality read ends (first base with Q-score < 20, corresponding to an error probability of 0.01, up to the 3’ end) and sequencing adapters were trimmed using custom java scripts. All datasets were pooled and assembled using Trans-ABySSv1.5.2 with multiple k-mers [28]. Potential coding sequences (CDSs) were predicted by TransDecoder (transdecoder.github.io). Redundant CDSs were clustered by CD-HIT-EST (v4.6) alignments using 95% sequence identity as the cutoff [29]. We used GhostKOALA to analyze all CDSs and determine their potential taxonomic affiliations [30]. Among the GhostKOALA annotations, we focused on CDSs affiliated with Metazoa and identified 148,052 such sequences. Of these, 48,000 showed affiliation with the poriferan reference species, *Amphimedon queenslandica*. These 148,052 CDSs were used as the reference transcriptome for the *M. grandis* host’s differential expression analysis.

### Differentially expressed gene (DEG) analysis

Short reads were aligned to aforementioned reference transcriptome using the burrows-wheeler alignment tool (BWA), v0.7.5a-r405 [31]. A count table was generated using SAMtools based on the BWA output. CDSs with a total count lower than 10 (less than 1 read per sample) were excluded before differential expression analysis [32]. The read counts of the remaining 139,578 CDSs were imported into DESeq2 in R environment [33]. Following the standard DESeq2 manual and filtering reads with FDR < 1%, the differentially expressed CDSs (hereafter DE CDSs) were identified for three comparisons between every two niches [33]. The blastp analysis and retrieval of Gene Ontology terms were performed in Blast2GO v3.2 using nr database with 10^− 5^ e-value [34]. The protein family annotations of DE CDSs were defined by InterProScan v5. RC7 with default parameters [35], using Pfam database as the primary annotating database, along with Gene3D, PRINTS, ProSiteProfiles, PANTHER, SMART, and SUPERFAMILY as complimentary databases. The visualization of GO terms associated with DE CDSs was carried out using REVIGO web server and the TREEMAP package in R environment (https://CRAN.R-project.org/package=treemap) [36]. The area of each rectangle in the treemap was proportional to the Benjamini-Hochberg (BH) corrected p-value, calculated using the ‘abs_log_pvalue’ option in REVIGO. Additionally, Pearson correlation coefficients were calculated to assess the relationship between DE CDSs and symbiotic microbial traits. The Pearson correlation coefficients were calculated between 1) normalized read numbers of each DE CDS and the Shannon index of functional gene diversity across nine samples, 2) normalized read numbers of each DE CDS and medians of the expression ratio across nine samples, These calculations were performed in Microsoft Excel 2016 using the ‘PEARSON’ function. Then a two-tail T-test was applied to define the significance of each *r* value. DE CDSs with a significant Pearson correlation (*P* < 0.01) to at least one of the factors (Shannon index of functional gene and/or median of expression ratio) were selected for further analysis.

## Results

### The rRNA pyrosequencing reveals uniform bacterial and fungal communities across three different niches

**Fig. 2:**
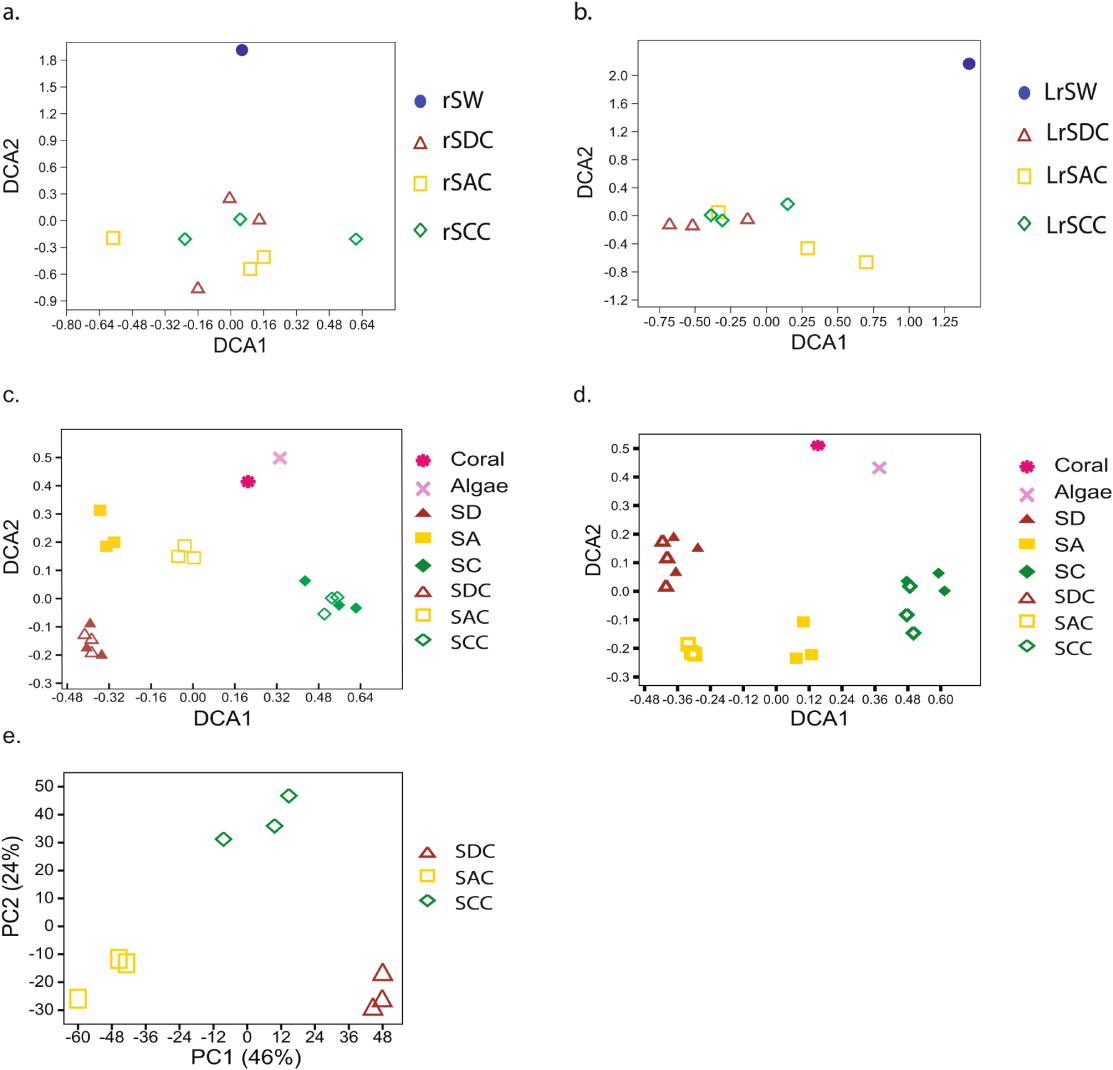
Illustration of niche-related pattern. Detrended Correspondence Analysis (DCA) on bacterial 16 S rRNA sequencing data (**a**) and fungal 28 S rRNA sequencing data (**b**); GeoChip bacterial datasets (**c**), and GeoChip fungal datasets (**d**). Principal Components Analysis (PCA) on sponge transcriptomic datasets (**e**). Code: SW– seawater; SA–*M. grandis* under alga *G. Salicornia*; SC–*M. grandis* on coral *P. compressa*; SD–*M. grandis* on rocks. If a code contains two letters, it means the data is derived from total DNA. Whereas if a code contains three letters, it means the data is derived from total cDNA. For example, SA means the data is derived from the total DNA of *M. grandis* when it grows under the algal mats. SAC mean the data is derived from the total cDNA of *M. grandis* when it grows under the algal mats

A total of 589 bacterial operational taxonomic units (OTUs) and 72 unique fungal OTUs were identified, with 94–247 bacterial OTUs and 32–37 fungal OTUs recovered from each sponge sample. One-way ANOVA indicated no significant differences in bacterial species richness and diversity across three different niches, while a slight variation was observed in fungal diversity (refer to Table S2). Taxonomic analysis showed 9 bacterial phyla and 11 fungal orders, with Betaproteobacteria and Capnodiales being the dominant taxa (Figs S1a and S1b). Notably, fungi exhibited low abundance and diversity in the sponge *M. grandis* compared with bacteria. Community structure comparisons revealed distinct separations between sponge-associated and seawater microbes, yet no niche-dependent clustering patterns were identified within sponge datasets (Fig. 2a and b). Weighted UniFrac distance matrix comparisons confirmed the uniformity of bacterial and fungal community structures across three different niches (Table S3).

At the OTU level, three Betaproteobacteria OTUs and one Capnodiales OTU dominated sponge datasets but were minimally represented in seawater datasets (< 2%), indicating the sponge-specificity of these microbes (Figs S1c and S1d). To distinguish closely related taxa, oligotyping analysis was performed on the reads from three predominant Betaproteobacteria OTUs and one predominant Capnodiales OTU. This revealed five oligotypes within the Betaproteobacteria OTUs without significant inter-niche variation (Bray-Curtis index-based one-way ANOSIM: *R* = 0.5007, *P* = 0.0604, permutation = 9999) (Fig. S2a and S2b). The Capnodiales OTU’s entropy analysis showed insufficient sequence variation, eliminating the need for further analysis (Fig. S2c).

### Metatranscriptome and geoChip microarray analyses uncover an inter-niche variability of *M. grandis* symbiotic bacterial and fungal gene expression

**Fig. 3:**
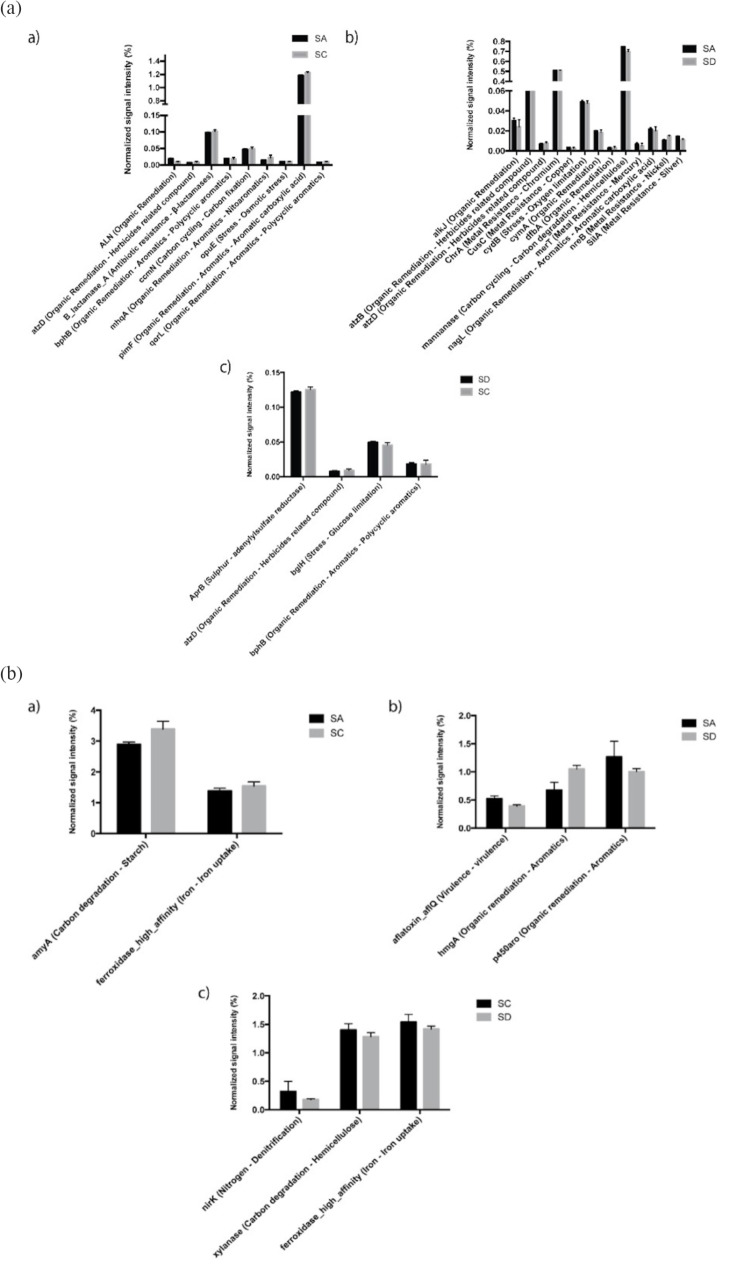
The relative abundance of bacterial (**a**) and fungal (**b**) functional genes are indicated by the normalized signal intensity (%). Genes with relative abundance significantly vary (*P* < 0.05) between different niches. Values are shown as the mean ± SE. The meaning of the codes is the same with those in Fig. 2

A total of 415 bacterial and 119 fungal genes associated with carbon/nitrogen/phosphorus/sulfur (C/N/P/S) metabolisms and stress response were identified (Table 1). Figure 2c and d illustrate the variability in genetic diversity and expression patterns, despite taxonomic stability. A core set of 370 bacterial genes, representing 89% of the identified sponge bacterial genes, were shared across three different niches, with only 23 showing minor variations in relative abundance (Fig. 3a). For fungi, 54% of the detected genes were considered to be the core genes, with a few exhibiting significant variabilities across three different niches (Fig. 3b). Functional gene categories displayed similar profiles across sponge, coral and algal datasets, with carbon degradation, organic remediation, and N/P/S transfer being predominant (Table 1). Bray-Curtis index based One-way ANOSIM (done in PAST 4.02) indicated the significant dissimilarity of core bacterial gene diversity at phylum-level (*R* = 0.6123, *P* = 0.0227 < 0.05, permutation = 9,999) and species-level (*R* = 0.4939, *P* = 0.0037 < 0.01, permutation = 9,999). In addition, the inter-niche variant dissimilarity of core fungal genes was significant at phylum-level (*R* = 0.5385, *P* = 0.0312 < 0.05, permutation = 9,999) and order-level (*R* = 0.7942, *P* = 0.0043 < 0.01, permutation = 9,999). This notion linked the niche-related separation to the inter-niche dissimilarity.

**Table 1:** Main functional categories and signal counts in GeoChip microarray

Bacterial category	SAC (8.85 ± 0.03)	SCC (9.36 ± 0.12)**	SDC (8.93 ± 0.02)*	Alga (9.42)	Coral (9.41)
**Carbon cycling**					
Acetogenesis	109.33 ± 5.13	176.33 ± 17.47	114 ± 0	155	159
Carbon degradation	2559.66 ± 46.3	4038.66 ± 449.05	2734.66 ± 63.21	3785	3583
Cellulose	231.66 ± 9.6	385 ± 39.23	245.66 ± 1.52	324	340
Chitin	503.66 ± 21.12	895.66 ± 88.11	529 ± 15.39	747	780
Hemicellulose	214.33 ± 5.13	347 ± 44.5	221.66 ± 6.42	317	307
Lignin	39.33 ± 2.08	60.66 ± 8.38	34.66 ± 0.57	56	57
Pectin	22 ± 0	23.33 ± 0.57	22 ± 0	3	2
Starch	1275.66 ± 36.29	1875.66 ± 214.64	1396.66 ± 29.73	1895	1662
Carbon fixation	280 ± 5.56	491.33 ± 49.81	291.33 ± 10.06	437	443
Methane	31.33 ± 3.05	42.33 ± 8.08	33 ± 1	38	43
**Nitrogen**					
Ammonification	181.33 ± 6.02	283 ± 41.58	193 ± 6.24	249	272
Anammox	NA	1.66 ± 0.57	NA	2	2
Assimilatory N reduction	58.33 ± 2.08	104 ± 14.73	58.66 ± 0.57	88	94
Denitrification	275.66 ± 13.2	529.66 ± 58.85	296.33 ± 6.8	430	492
Dissimilatory N reduction	69 ± 2.64	111.66 ± 10.4	70 ± 3	90	102
Nitrification	124 ± 6.55	197 ± 23.38	135 ± 1.73	180	185
Nitrogen fixation	163.66 ± 4.5	243.66 ± 29.26	179.33 ± 4.5	239	228
**Organic remediation**					
Aromatics	429 ± 18.52	970.66 ± 142.69	476 ± 13.45	805	807
Chlorinated solvents	30 ± 1	68.66 ± 11.01	34.66 ± 1.52	55	55
Herbicides	46.33 ± 2.51	111.33 ± 17.95	48.66 ± 0.57	74	85
Other Hydrocarbons	31.66 ± 2.51	72.66 ± 8.38	31 ± 2	57	61
Pesticides	23 ± 1.73	41.66 ± 8.38	21.66 ± 0.57	40	35
**Phosphorus**	359.33 ± 18.71	638 ± 69.31	383.66 ± 9.45	505	587
**Stress**					
Cold shock	2 ± 0	5.33 ± 2.88	2 ± 0	4	4
Glucose limitation	3 ± 0	6.66 ± 0.57	3 ± 0	5	5
Heat shock	45 ± 2	88.66 ± 15.3	54 ± 2	71	83
Nitrogen limitation	33.33 ± 2.08	81 ± 14.79	41.66 ± 2.08	69	68
Osmotic stress	11.66 ± 1.52	29 ± 2.64	15 ± 1	25	25
Oxygen limitation	36.66 ± 0.57	73.33 ± 12.42	40.33 ± 2.88	69	63
Oxygen stress	104.33 ± 5.85	257 ± 55.43	129.66 ± 0.57	194	203
Phosphate limitation	121 ± 2.64	259.33 ± 52.44	150.66 ± 3.51	194	217
Protein stress	15.66 ± 0.57	29.66 ± 3.21	17 ± 0	24	23
Radiation stress	31 ± 0	64.66 ± 10.21	35.33 ± 2.88	46	52
σ-factors	103.66 ± 5.13	254 ± 34.87	116 ± 5.29	196	207
**Sulphur**					
Adenylylsulfate reductase	36.66 ± 0.57	77.33 ± 5.03	39.66 ± 2.08	61	66
Sulfite reductase	297.66 ± 5.77	514 ± 63.23	315.33 ± 8.02	446	442
Sulphur oxidation	114 ± 5.56	183.33 ± 24.58	118.66 ± 5.85	152	179
**Fungal category**	**SAC** **(**5.3 ± 0.04**)**	**SCC** **(**6.06 ± 0.11)**	**SDC** **(**5.39 ± 0.02**)**	**Alga** **(**6.93**)**	**Coral** **(**6.81**)**
**Carbon degradation**					
α-galactoside	3.33 ± 0.57	5.33 ± 2.88	5 ± 1.73	9	14
Cellulose	24 ± 1	47 ± 9.64	19.33 ± 1.15	102	99
Chitin	16.33 ± 2.88	29.33 ± 5.68	12.33 ± 0.57	75	62
Cuitin	1 ± 0	4.66 ± 2.3	2 ± 0	7	2
Glucan	3.33 ± 0.57	6.33 ± 0.57	1 ± 0	18	15
Hemicellulose	8 ± 0	27.33 ± 4.72	13 ± 1	53	59
Inulin	NA	1 ± 0	2 ± 0	2	3
Lignin	49.33 ± 3.51	111.33 ± 21.96	65.66 ± 3.05	242	227
Pectin	1 ± 0	4.33 ± 0.57	1.66 ± 0.57	10	9
Polygalacturonate	6.66 ± 0.57	19.33 ± 2.88	8 ± 1	36	27
Starch	15.33 ± 1.52	28 ± 3.46	16 ± 1	64	56
**Chitin synthesis**	17 ± 1	24.33 ± 3.05	21 ± 1.73	60	56
**Iron**					
Iron transport	2.33 ± 0.57	5.33 ± 0.57	2 ± 0	10	7
Iron uptake	5 ± 1	16.33 ± 2.88	6.66 ± 0.57	33	39
Siderophore transporter	1 ± 0	3.66 ± 0.57	1 ± 0	8	7
Siderophore synthesis	1.66 ± 0.57	1 ± 0	1 ± 0	5	5
**Nitrogen**					
Ammonification	1.33 ± 0.57	3.33 ± 0.57	2.66 ± 0.57	9	7
Denitrification	4.66 ± 1.15	9.66 ± 2.3	3 ± 0	20	16
**Organic Remediation**					
Aromatics	51.66 ± 2.3	111 ± 22.6	58.66 ± 1.15	216	208
**Phosphorous**					
Phosphorus utilization	6.33 ± 0.57	11 ± 2	5.33 ± 0.57	25	22
**Stress**					
Heat shock	1 ± 0	4 ± 1.73	1.66 ± 0.57	13	6
Nitrogen limitation	1.66 ± 0.57	9 ± 2.64	NA	15	13
Oxygen stress	10.66 ± 0.57	19 ± 3.46	12 ± 1	38	38
Phosphate limitation	1 ± 0	1.66 ± 0.57	NA	7	7
**Sulphur**					
Sulfate transfer	1 ± 0	7.66 ± 2.3	1 ± 0	14	11
Sulfite reductase	6 ± 0	6.66 ± 0.57	5 ± 0	12	11

*Note* The meaning of the codes is the same with those in Fig. 2. The signal counts of sponge DNA datasets (SA, SD, and SC) are the same with the cDNA datasets, as only the spots with both DNA and cDNA signals are considered to be positive. For sponge datasets, values are shown as mean ± SD (*n* = 3). Shannon indices based on all positive signals are shown in brackets *P < 0.05, ** *P <* 0.01

**Fig. 4:**
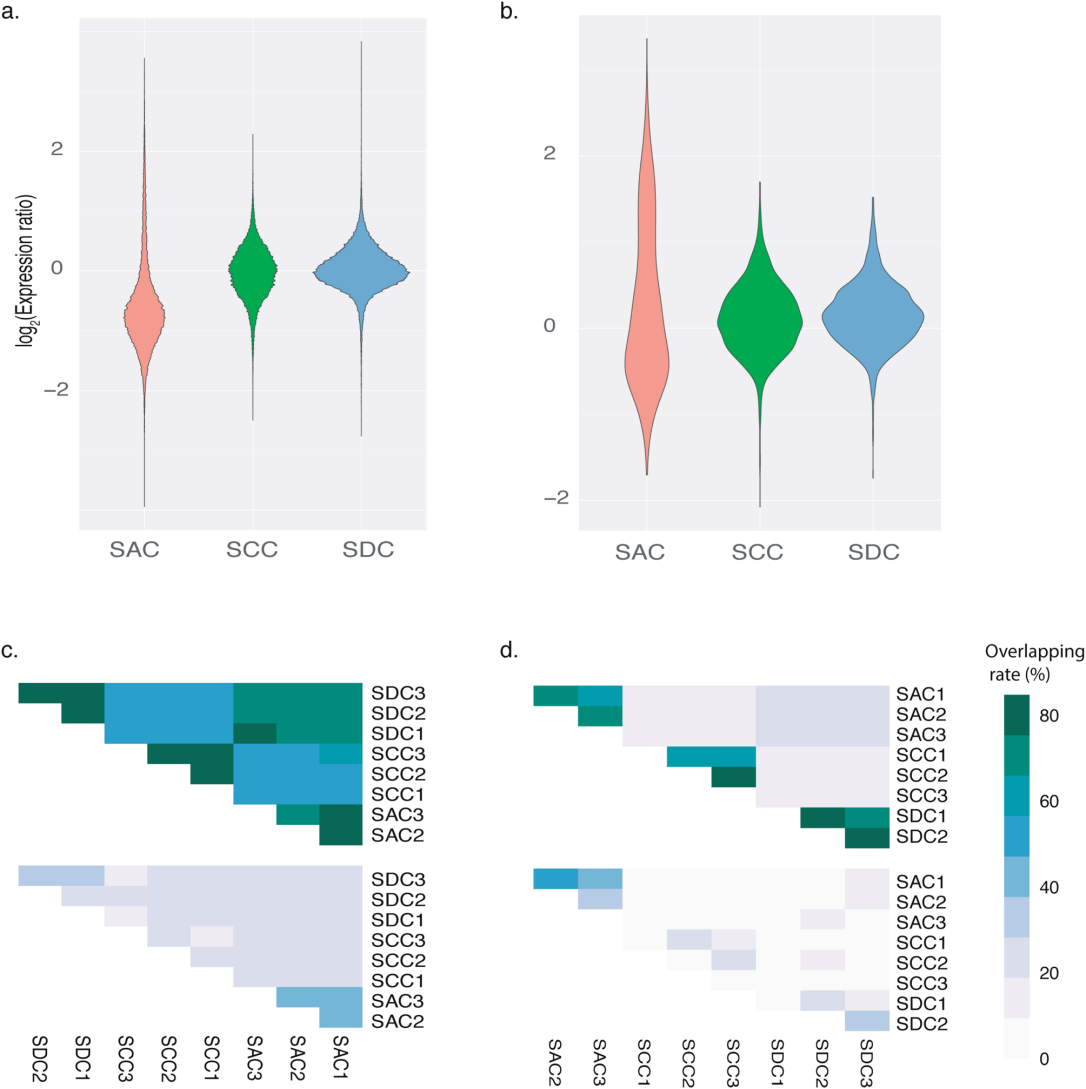
Illustration of inter-niche microbial functional gene expression pattern. Violin plot depicting the distribution of expression ratio of bacteria (**a**) and fungi (**b**). Overlapping pattern of positive signals (upper) and high-expression-ratio signals (lower) from bacteria (**c**) and fungi (**d**). The meaning of the codes is the same with those in Fig. 2

**Fig. 5:**
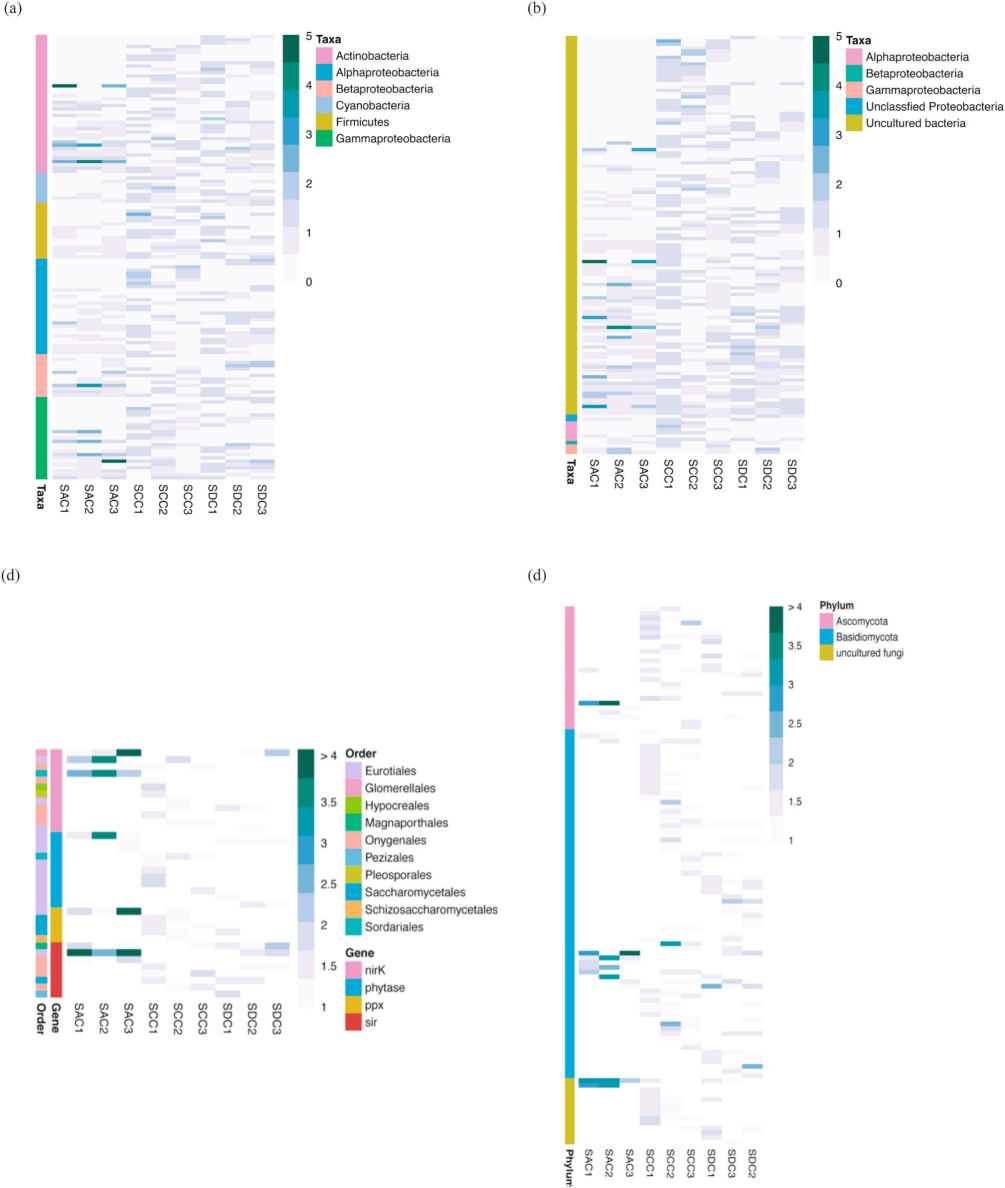
Distribution of high-expression-ratio variants in bacterial genes *ure*C (**a**), *nir*K/S (**b**), and fungal genes in N/P/S cycling (**c**), phenol oxidase (**d**). More results please see Fig. S4. The meaning of the codes is the same with those in Fig. 2

The expression ratio was calculated to reflect the transcriptional richness of a certain variant, i.e. cDNA signal density/DNA signal intensity. Community-level comparisons demonstrated that when *M. grandis* grew under algal mats (SAC), the dispersion degree became larger, and the median of expression ratios was significantly lower (One-way ANOVA: *P*_bacteria_=0.0023 < 0.01; *P*_fungi_=0.0012 < 0.01) (Fig. 4a and b). As shown in Fig. 4c, the intra-overlapping rate of positive bacterial variants were $\sim$ 80% and the inter-overlapping rate ranged from 50 to 80%. Whereas for high-expression-ratio variants, the intra-niche overlapping rate was generally below 50% and the inter-niche overlapping rate was generally lower than 40%. Similar trend with lower overlapping rates was observed in the comparisons of fungal datasets, with the inter-niche overlapping rate of high-expression-ratio variants dropped under 20% (Fig. 4d). The inter- and intra-niche variability of high-expression-ratio variants were prevalent, we could hardly find a certain variant with stable intra-/inter-niche expression ratio. Genes related to the major geochemical cycles (C/N/P/S) were shown in Fig. 5 and S4. Consequently, the inter-niche variability of high-expression-ratio variants was obvious, particularly sponge *M. grandis* under alga *G. salicornia* were significantly different from the other two niches.

### Variability of sponge host’s gene expression profiles across three different niches

We detected 22,908 DE CDSs from three pairwise comparisons of the host transcriptome. About 36% (8,283) DE CDSs had non-ambiguous blast hit (neither ‘hypothetical’ nor ‘predicted’ proteins), about 22% (5,147) DE CDSs had protein family annotations, and about 20% (4,544) DE CDSs had GO annotations. The ordination analysis elucidated a clear separation among three groups (SDC, SAC and SCC) of sponge hosts (Fig. 2e), in which the niche type (PC1) explained 46% of the variance. Gene ontology treemaps demonstrate that the host’s biological processes vary between different niches (Fig. S5). For example, compared with *M. grandis* on rocks, the response stress and carbohydrate metabolism related genes were upregulated for *M. grandis* under alga *G. salicornia*, the response stress and glucosinolate catabolism related genes were upregulated for *M. grandis* on the surface of coral *P. compressa*. These results, interesting but not unexpected, implied that the sponge hosts were under different physiological status and might cope with different biotic and abiotic stimulus.

**Fig. 6:**
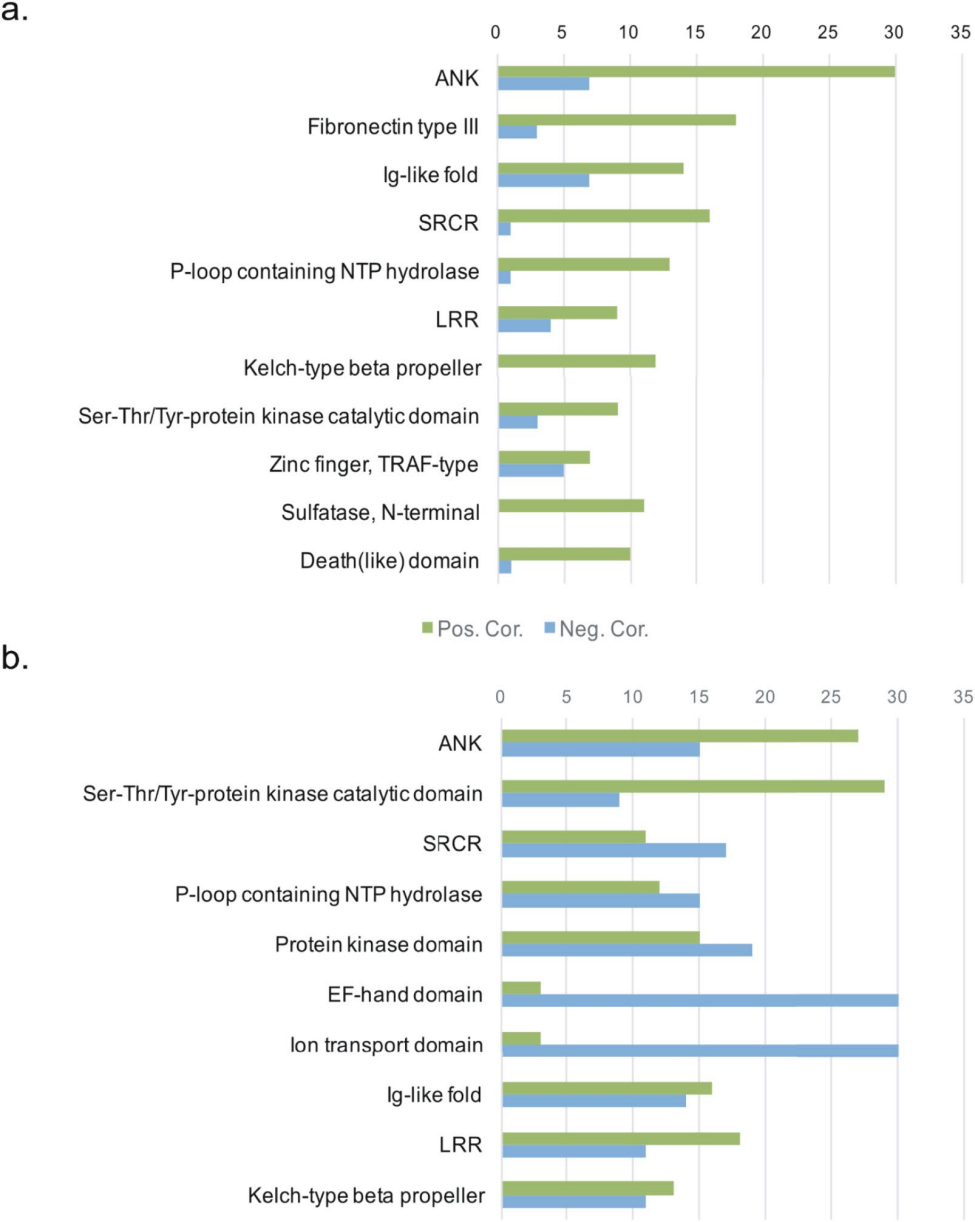
The top protein families containing the most DE CDSs correlated with the symbiont functional gene diversity (**a**) and expression ratio median (**b**). Green: positively correlated; Blue: negatively correlated

To provide insights into the host-microbe interplay, we calculated the correlation pattern between the sponge host DE CDSs and microbial symbiont traits. There were 2804 DE CDSs correlating with the variation of gene expression ratio medians (*p* < 0.01), including 1156 DE CDSs with protein family annotation, encompassing 380 protein families. Based on Pearson correlation coefficients, we identified 972 DE CDSs that correlated with the variation of functional gene shannon indexes (*p* < 0.01). Figure 6 shows the top major protein families correlated with the microbial functional gene diversity and expression ratio changes, e.g. ankyrin repeat (ANK), Leucine-rich repeat (LRR) and Scavenger Receptor Cysteine-Rich (-like) domain (SRCR). Most of these protein families are positively correlated to microbial functional gene diversity and expression ratio changes, e.g. ANK, SRCR, LRR, while few e.g. EF-hand domain and ion transport domain show negative relationship.

## Discussion

### Niche–dependent sponge hologenome expression profiles: host and its bacterial/fungal symbionts

Though environmental factors can affect the microbial community to some extent, in general, the core microbial community of a sponge species is relatively stable over different habitats, because of the host’s role in determining its symbiotic microbial community [3, 9, 10]. In the case of *M. grandis*, our previous DNA-based studies illustrated that *M. grandis* had relative consistent bacterial/fungal community [15, 16]. Compared with these DNA-based sponge microbial community analysis, the RNA-based approach is deemed to be more sensitive to the host physiological and environmental impacts. In this study, according to RNA-based 16 S rRNA and 28 S rRNA sequencing analysis, no significant difference in bacterial and fungal richness/diversity was observed for *M. grandis* across all the three different niches (Fig. 2 and S1). This result supported the notion that sponge host plays a key role in determining the complexity of symbiotic microbial communities regardless the different habitat types [3].

GeoChip microarray, a comprehensive microarray, has been developed and applied to study functional diversity and metabolic potential [37]. Probes on the GeoChip, which are species-specific or group-specific, allowed assessment of inter-niche difference at the variant level. The extensive and specific probe sets on GeoChip provide a fine resolution for decoding the functions and activities of *M. grandis* microbiome. In this study, GeoChip, together with metatranscriptome analysis, uncovered niche-dependent functional gene diversity and expression pattern of bacterial and fungal symbionts in three different niches (Figs. 3, 4 and 5 and S3-4; Table 1), indicating an inter-niche variability of *M. grandis* microbiome to niche response/adaptation.

To date, the sponge host’s metabolism and metabolic change in different niches are rarely known. It is the first time to investigate the sponge host’s gene expression profiles under different niches in this study. The niche-dependent *M. grandis* host transcriptome indicates sponge host’s different physiological status in three different niches (Fig. S5). Totally, the sponge hologenome (host and microbiome) functional profiles comparison indicates the response/adaptation of *M. grandis* holobiont to different niches. For instance, the functional gene expression ratio in *M. grandis* under alga *G. salicornia* (SAC) was significantly lower compared with the other two niches (Fig. 4a and b), this might result from the stress from the alga *G. salicornia e.g.* shielding of sunlight, lower pH, and drastic changes of dissolved oxygen [38]. According to the morphological features, *M. grandis* under the algal mats had smaller ostia and thinner tissues compared to the other two niches (data not shown). The up-regulated gene expression found in the SAC sponge host, particularly the hypoxia, apoptotic process, and polysaccharide catabolic process related categories, are likely the reflection of interplay between *M. grandis* and these stress factors. Hypoxia can stimulate the oxidant production and consequently induce DNA damage [39], which is possibly related to the observation of up-regulated spliceosomal tri-snRNP complex assembly in this study. The up-regulation of polysaccharide catabolic metabolism implied *M. grandis* host was utilizing alternative carbon sources to compensate for the energy supply. The up-regulation of apoptotic process might also reflect the tendency of reducing the energy cost. As for SDC sponge datasets, the most distinguishable up-regulated GO-term category was mechanical stimulus. It was potentially related to the relatively higher water impact, which could affect the pressure equilibrium of incurrent and excurrent canals, irritating the beating of flagella, even clogging the canals. Demosponges, including *Mycale*, can contract the canals and close their ostia and oscula to gain control over the flow gradually. In the case of *M. grandis* on the surface of coral *P. compressa* (SC/SCC), the diversity of bacterial and fungal genes related to C/N/P/S and stress was the highest compared with the other two niches (Table 1), indicating the possible competition with coral *P. compressa*.

### Sponge-microbes interplay in different niches: host’s innate immune and microbial recognition

Two key areas of the sponge-microbes symbioses are the molecular determinants of sponge-microbe interactions and how sponge innate immunity mediates the sponge microbiome [4]. The availability of a nearly complete genome for the sponge *Amphimedon queenslandica* has provided insights into how the host innate immune system may contribute to holobiont interactions [1]. Pattern recognition receptors (PRRs) are proteins expressed by cells of the host’s innate immune system. PRRs are thought to identify microbe-associated molecular patterns and recognize microbial ligands encountered within the holobionts. When a PRR binds to a microbial ligand, it can set off a signal transduction cascade that results in the transcription of immune response genes encoding products with antibacterial activity [4]. PRRs from sponges, especially nucleotide-binding domain and Scavenger Receptor Cysteine-Rich (SRCR)-like domains, are thought to be the key innate immune factors mediating sponge-microbe recognition [40]. A recent study on three sponge hologenomes showed that the SRCR domain is expanded in a low-microbial-abundance sponge (*Stylissa carteri*) compared with a high-microbial-abundance sponge *Xestospongia testafinaria* [41]. The up-regulation of SRCR domain containing genes in the sponge *Petrosia ficiformis* was suggested to be in relation to the recognition of cyanobacteria [42]. Eukaryotic-like proteins (ELPs) from sponge microbial symbionts have been illustrated as important factors mediating the amoebal phagocytosis [43], i.e. microbes with ELPs could probably escape the phagocytosis by the sponge hosts. N-terminal Ankyrin repeat (ANK) and Leucine-rich-repeat (LRR) are prevalent in eukaryotes and involved in a wide range of protein-protein interaction and cell communication [44, 45]. In addition to ANK and LRR, sponge genes related to cell adhesion and auto/allo-recognition, such as the immunoglobulin domain, were found to be present in the extracellular part of the receptor tyrosine kinase in the sponge *Geodia cydonium* [46].

This study identified 33 DE CDSs encompassing innate immunity and adhesion correlated with symbionts’ functional genes (Figs. S6 and S7). The expression of genes encoding protein families related to innate immunity and microbial recognition, e.g. SRCR, ANK and LRR, changed with the niches and appeared to be highly correlate with the microbial attributes (Figs. 6 and S6-S7). Other protein families such as P-loop containing nucleoside triphosphate (NTP) hydrolase, Kelch-type beta propeller, and Ser-Thr/Tyr-protein kinase catalytic domain were found in a wide spectrum of biological processes [21, 47]. The detected differential expression of those protein families (Fig. 6), together with the host’ intracellular signal transduction-related gene expression changes (Fig. S5), could affect the host-microbe interaction via multiple signaling pathways and/or metabolic interactions. Though it is difficult to link the specific biological processes to the variations of these functional gene diversity and transcriptional richness, these results underscore the importance of these proteins in sponge-microbe interaction and potential critical roles in the regulation/selection of symbionts. Particularly, the fact that the expression profiles of *M. grandis* host’s genes related to innate immunity are closely correlated with the genes’ diversity and transcriptional richness of symbiotic microbes indicates the close and dynamic host-microbes association.

## Conclusion

In this study, the RNA-based analysis indicates *M. grandis* has largely similar bacterial and fungal communities across different niches. However, GeoChip microarray and metatranscriptome analyses reveal niche-dependent functional gene diversity and transcriptional richness of bacterial and fungal symbionts. Furthermore, niche-related physiological status of sponge host is implied since its gene expression patterns are niche-dependent. Notably, the innate immunity of the sponge host is implied to be closely related to the diversity and transcriptional richness of its symbiotic microbes. Of course, these findings need to be echoed by more species of sponges with different phylogenetic and biogeographical profiles since this report is only a case of *M. grandis* holobiont in three different niches. Even so, the results from this study support the hypothesis that the gene expression and interplay of host-microbes of a specific sponge species are niche-dependent though the symbiotic microbial community is relative stable, providing novel insights into the black box of sponge holobionts at the hologenome level. The niche-related sponge holobiont metabolic profiles and host-microbes interplay is helpful for our understanding of the adaptation of encrusting sponge *M. grandis* to different niches.

### Electronic supplementary material

Below is the link to the electronic supplementary material.

Supplementary Material 1

## Data Availability

The 454 sequencing datasets are deposited in European Nucleotide Archive with accession number PRJEB12741. The RNA-Seq raw data are available under NCBI BioProject ID PRJNA317402.
