# Supplementary material for: Niche–dependent sponge hologenome expression profiles and the host-microbes interplay: a case of the hawaiian demosponge Mycale Grandis
Source: Environ Microbiome. 2024 Apr 8;19:22. doi: 10.1186/s40793-024-00563-8 (PMC11000336; doi:10.1186/s40793-024-00563-8)
Supplement: Supplementary file 1 — Supplementary Material 1 [file 40793_2024_563_MOESM1_ESM.docx]

**Supplementary Information**

**Niche–dependent gene expression profiles and the host-microbes interplay of sponge holobionts: a case of the Hawaiian demosponge *Mycale grandis***

Fang Liu^1,2^, Taewoo Ryu^3^, Timothy Ravasi^3^, Xin Wang^4^, Guangyi Wang^5^, Zhiyong Li^1,2^*

^1^State Key Laboratory of Microbial Metabolism, School of Life Sciences & Biotechnology, Shanghai Jiao Tong University, Shanghai, 200240, P. R. China

^2^Joint International Research Laboratory of Metabolic & Developmental Sciences, Shanghai Jiao Tong University, Shanghai, 200240, P. R. China

^3^Marine Climate Change Unit, Okinawa Institute of Science and Technology Graduate University (OIST), Okinawa, 904-0495, Japan

^4^Department of Microbiology, Miami University, Oxford Ohio, 45056, USA

^5^School of Environmental Science and Engineering, Tianjin University, Tianjin, 300072, P. R. China

***Correspondence:**

Zhiyong Li: zyli@sjtu.edu.cn


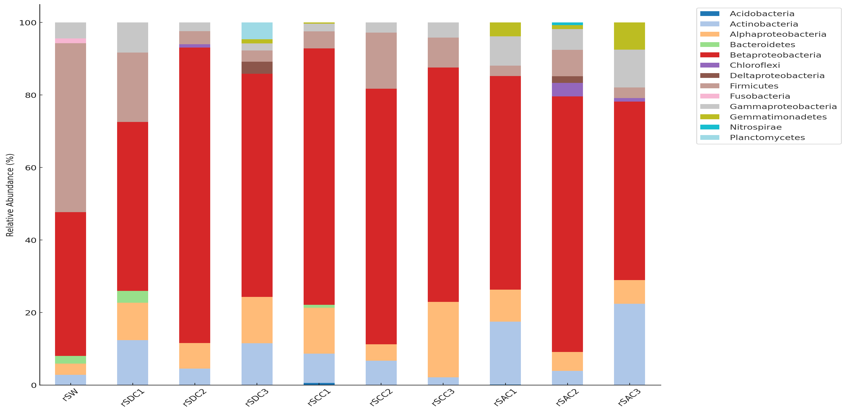
a

b


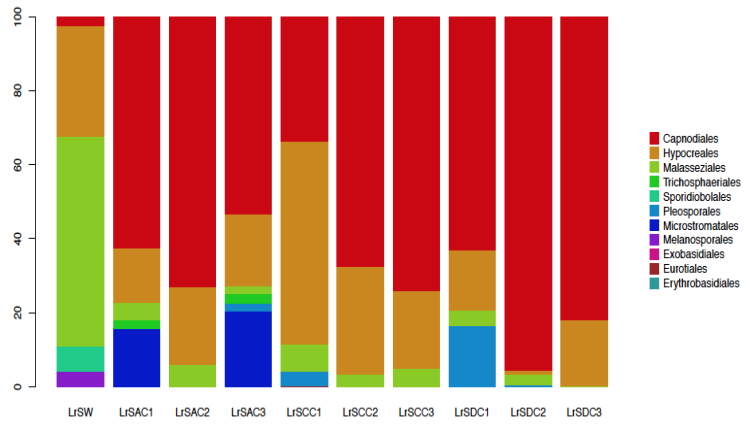
c d

**Fig. S1.** The phylogenetic composition of bacterial (a) and fungal (b) communities is represented on the y-axis as the relative abundance of normalized reads. Heatmaps illustrated the distribution of bacterial (**c)** and fungal (**d**) OTUs (OTUs with relative abundance ≥1% in each dataset were selected). Note: SW – seawater; SA –*M. grandis* under alage *G. Salicornia*; SC –*M. grandis* on coral *P. compressa*; SD –*M. grandis* on rocks; The C after SD, SA, SC means cDNA sample.


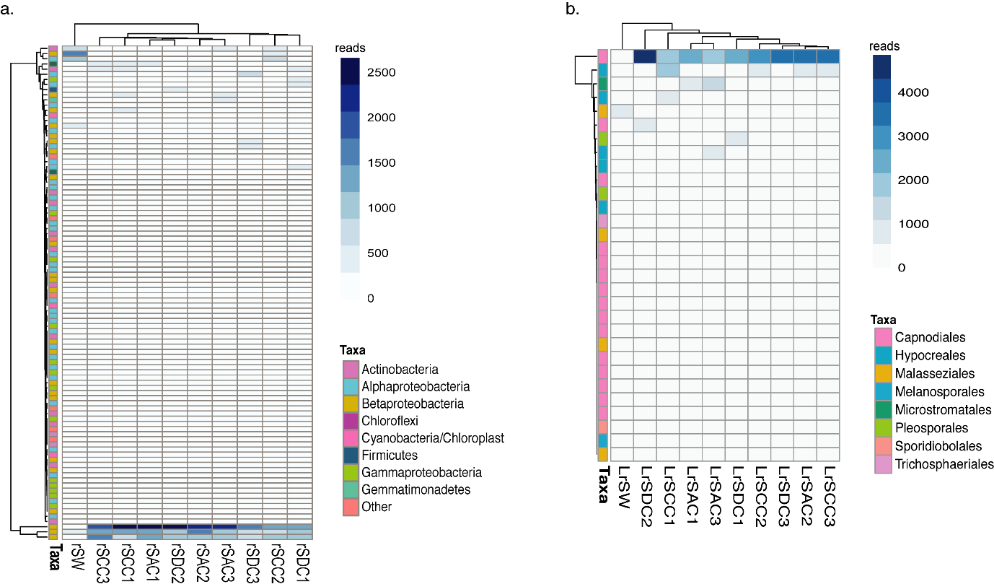

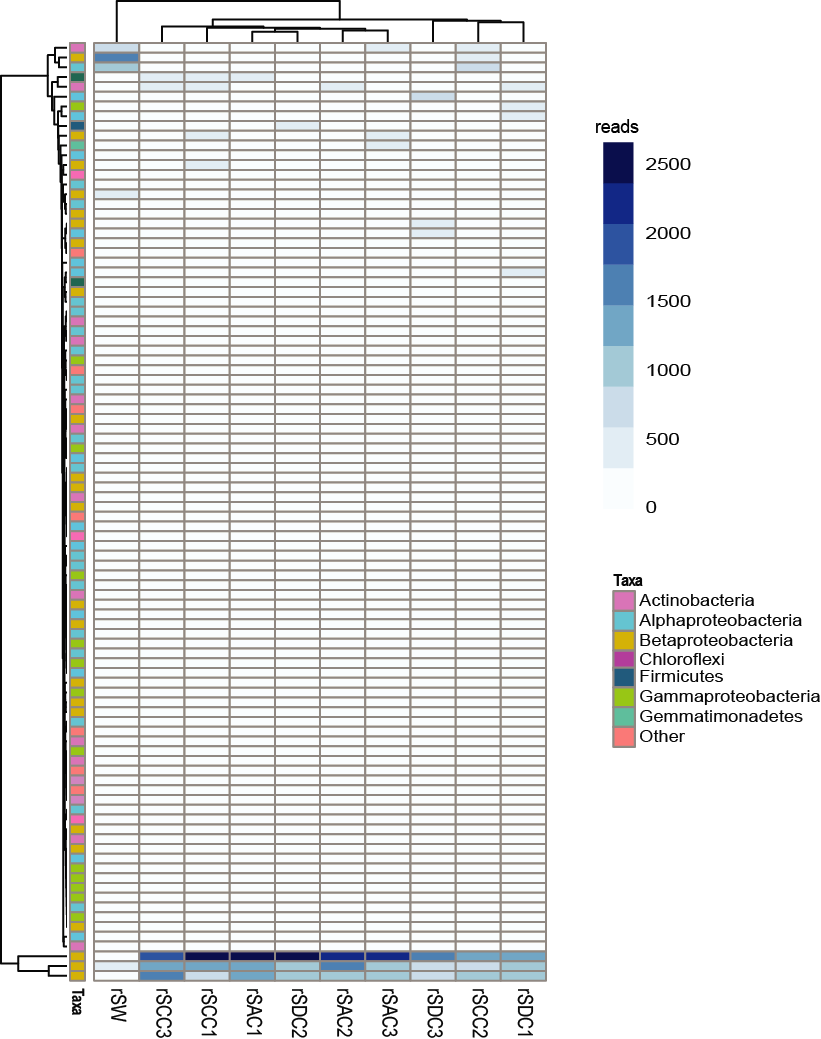


**
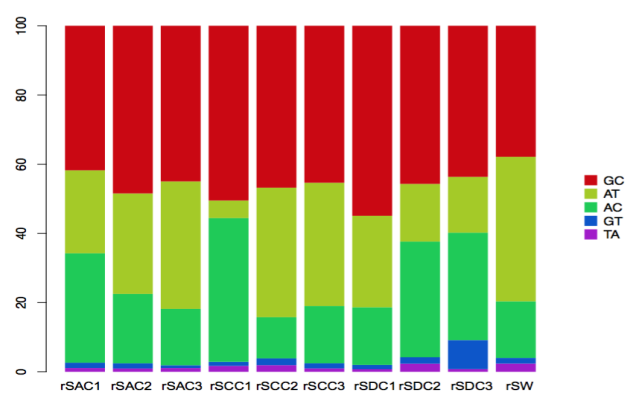
**a b


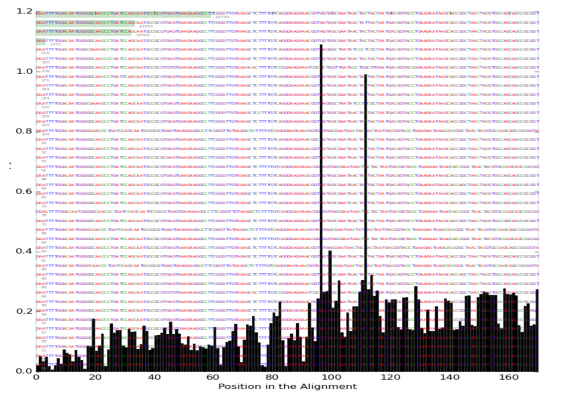


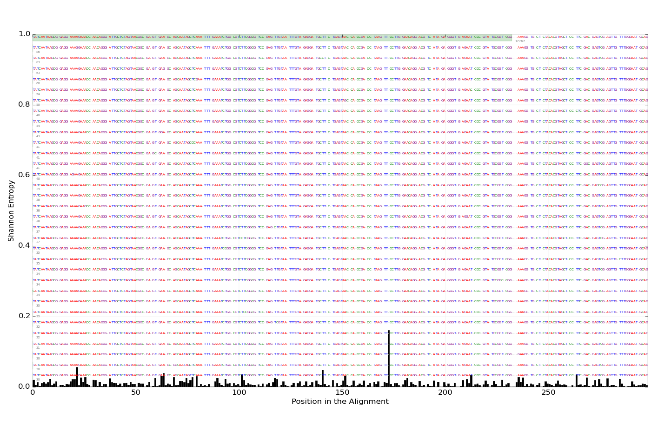
c

**Fig. S2**. Results of oligotyping analysis on predominant bacterial and fungal OTUs. **a**) Entropy analysis of sequences of the predominant bacterial OTU, position 93 and 116 were chosen due to entropy values >0.4; **b**) Relative abundance of identified oligotypes across samples; **c**) Entropy analysis of sequences within the predominant fungal OTU, no need to further dissect as no position with entropy value >0.2. Note: SW – seawater, SAC –*M. grandis* under alga *G. salicornia*; SCC - *M. grandis* on coral *P. compressa*; SDC - *M. grandis* on the rocks. The C after SD, SA, SC means cDNA sample.


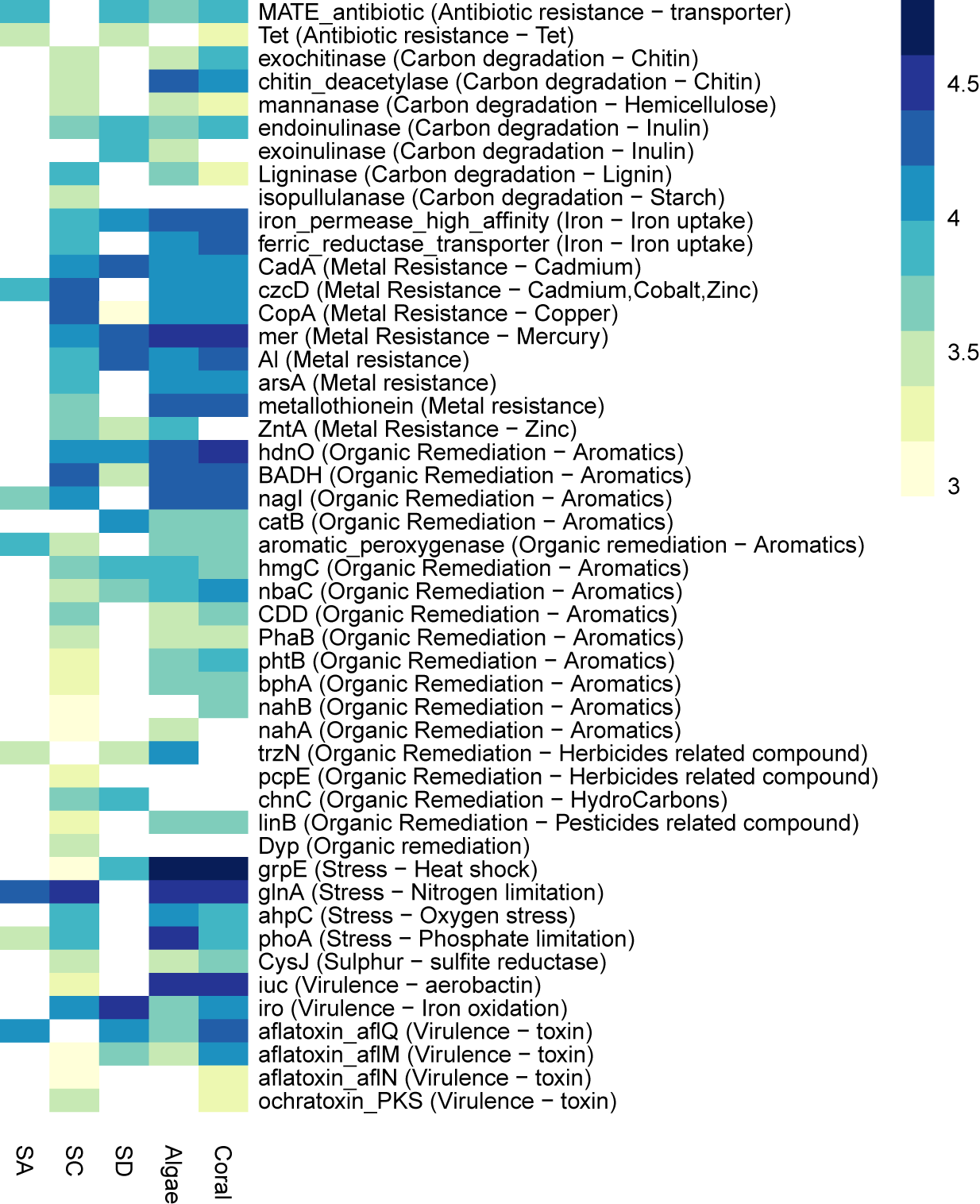


**Fig. S3.** Distribution of variable fungal genes. Gene categories and sub-categories (if available) were shown in parenthesis. Means of normalized signal intensity (n=3) were showed after log_10_ transformation. Note: *M. grandis* on the rocks by the dock (SD), *M. grandis* on coral *P. compressa* (SC), *M. grandis* under algae *G. salicornia* (SA).


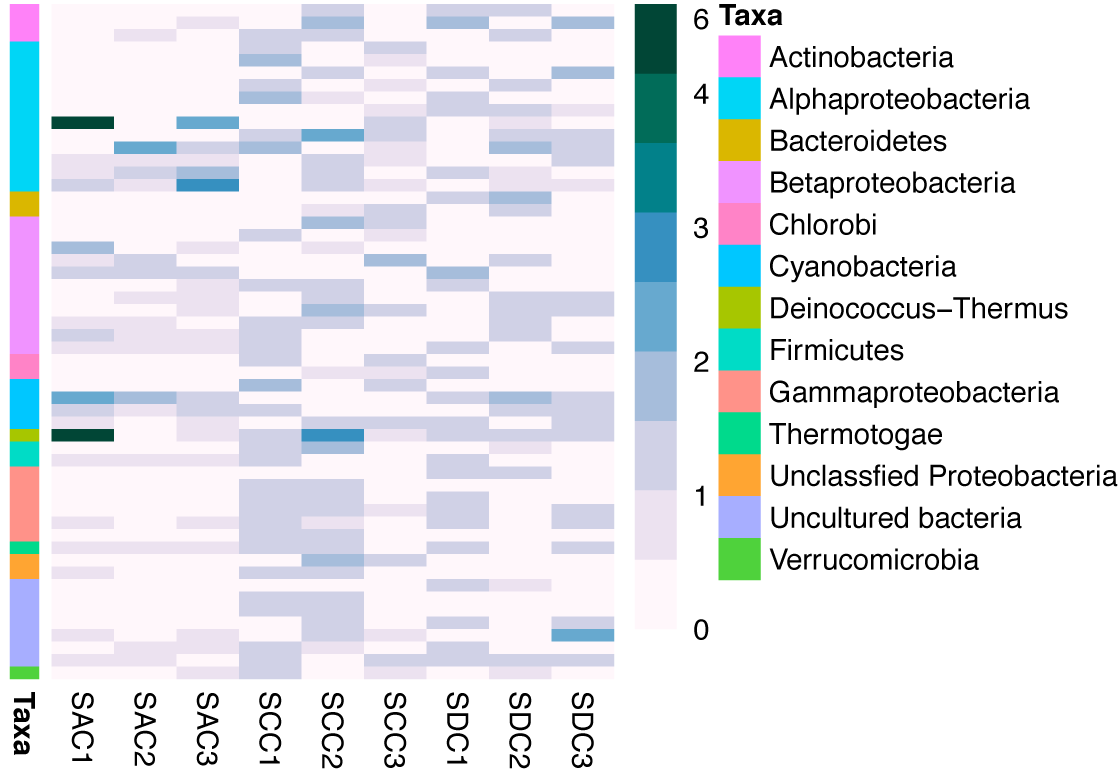
a


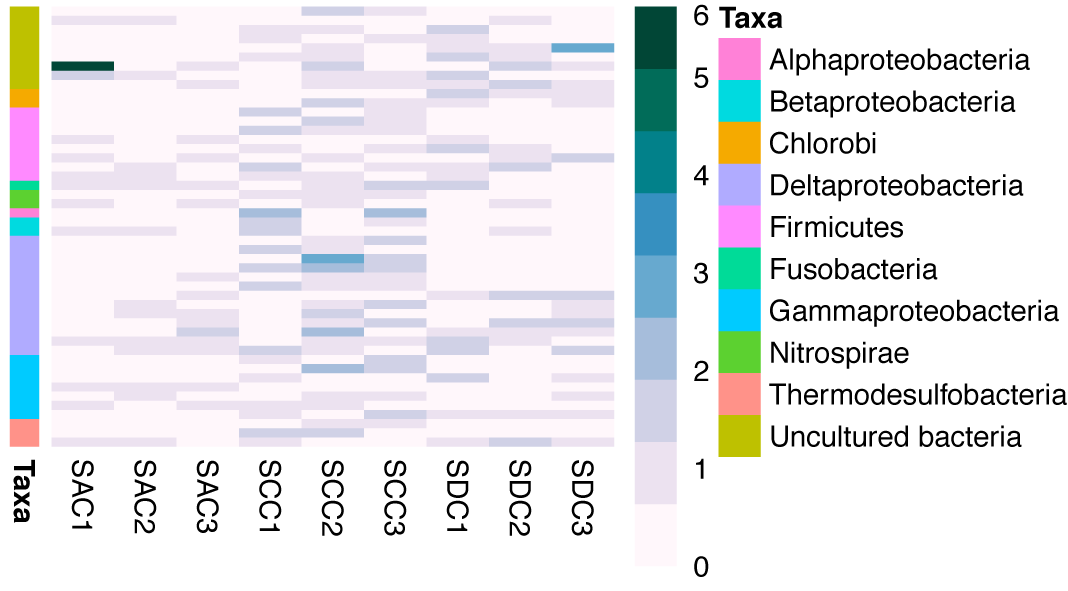
b


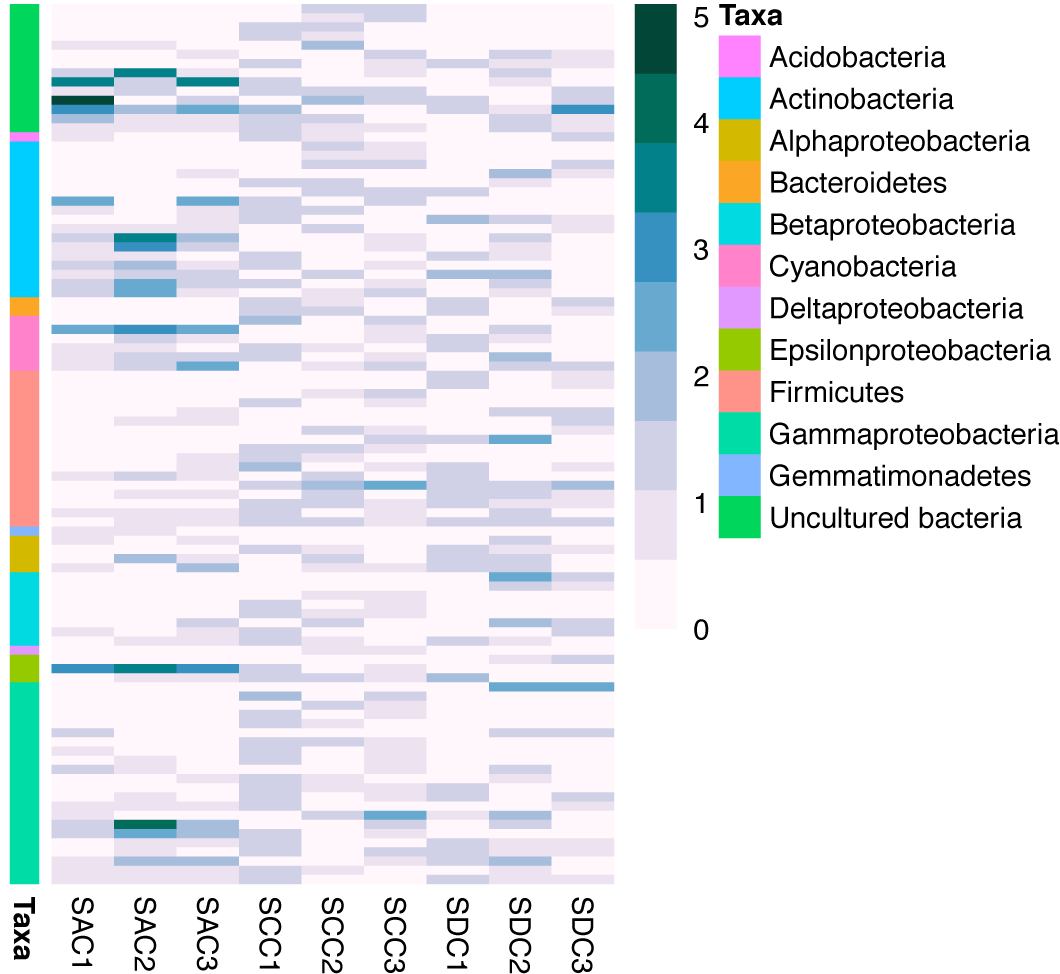
c

**Fig. S4.** Distribution of high-expression-ratio variants in bacterial and fungal communities. Bacterial RuBisCO gene (**a**), *apr*A/B gene (**b**), and *ppk* gene (**c**), respectively. Note: SAC –*M. grandis* under alga *G. salicornia*; SCC - *M. grandis* on coral *P. compressa*; SDC - *M. grandis* on the rocks. The C after SD, SA, SC means cDNA sample.


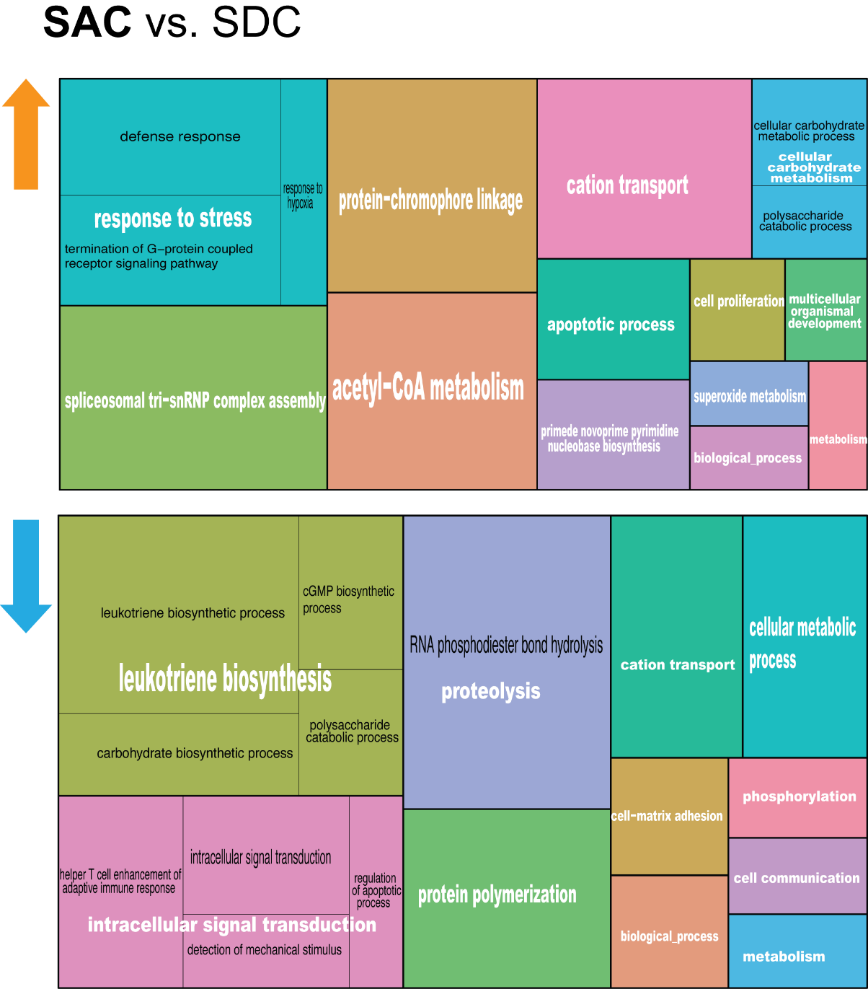

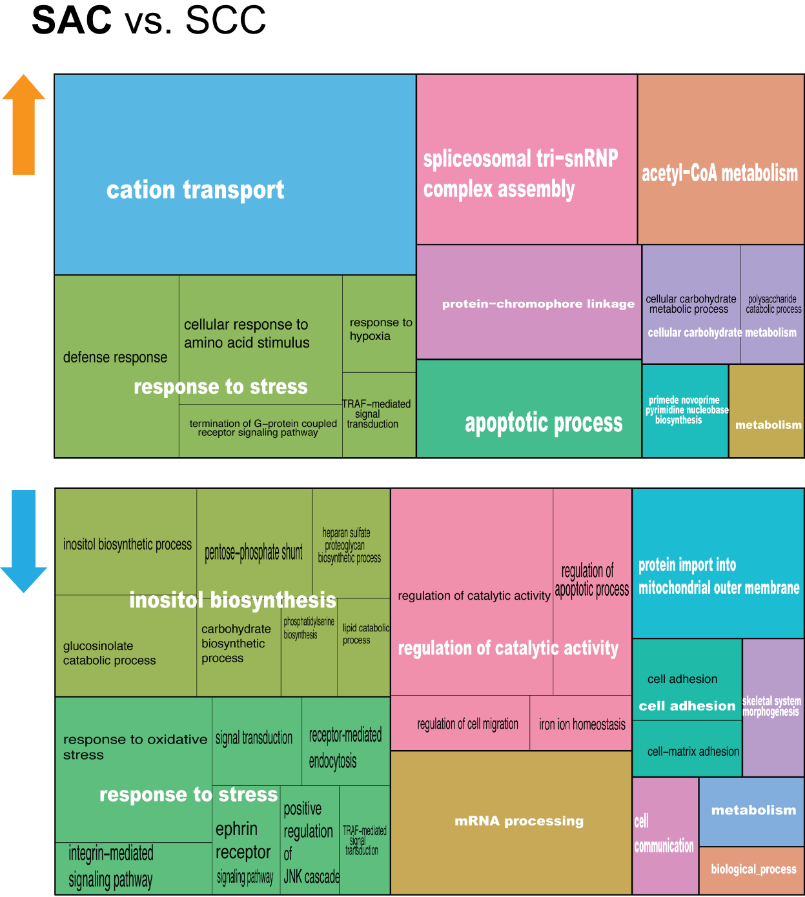
a

b

C


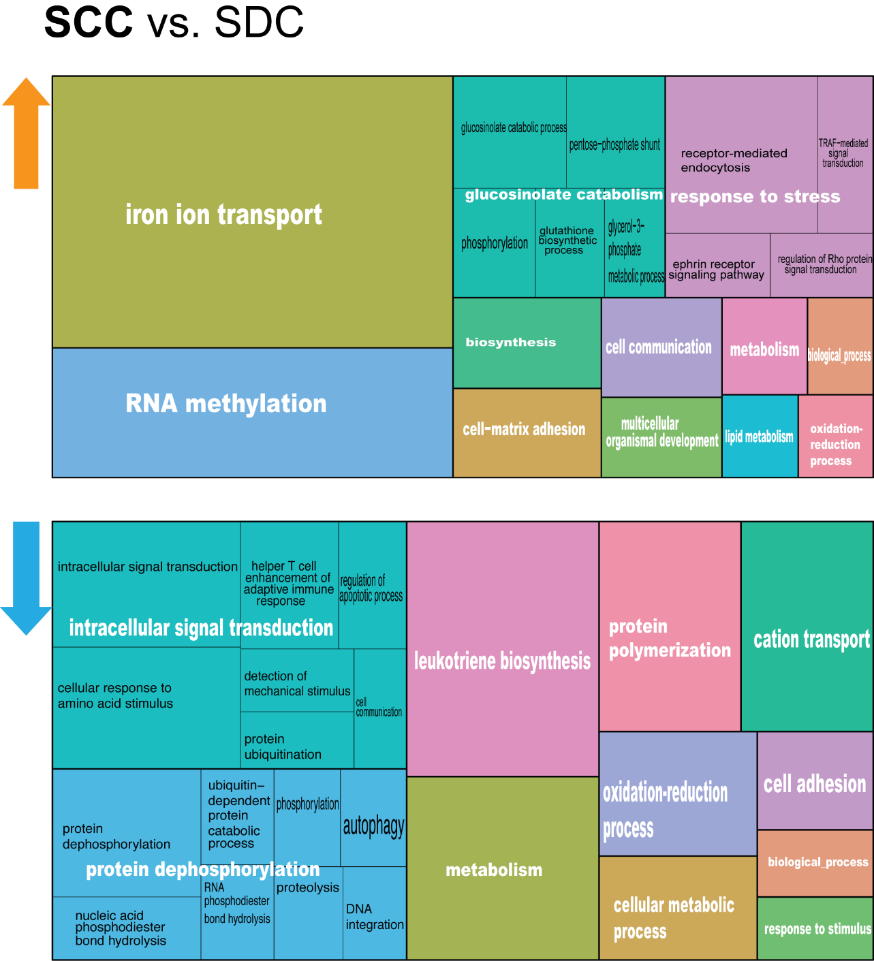


**Fig. S5.** Gene Ontology treemaps for annotated sponge host’s differentially expressed genes (a-SAC vs. SDC, b-SCC vs. SDC, c- SAC vs. SCC). Bold letters indicated the niche where genes were up- (yellow arrow) or down- (blue arrow) regulated. Note: SAC –*M. grandis* under alga *G. salicornia*; SCC - *M. grandis* on coral *P. compressa*; SDC - *M. grandis* on the rocks. The C after SD, SA, SC means cDNA sample.


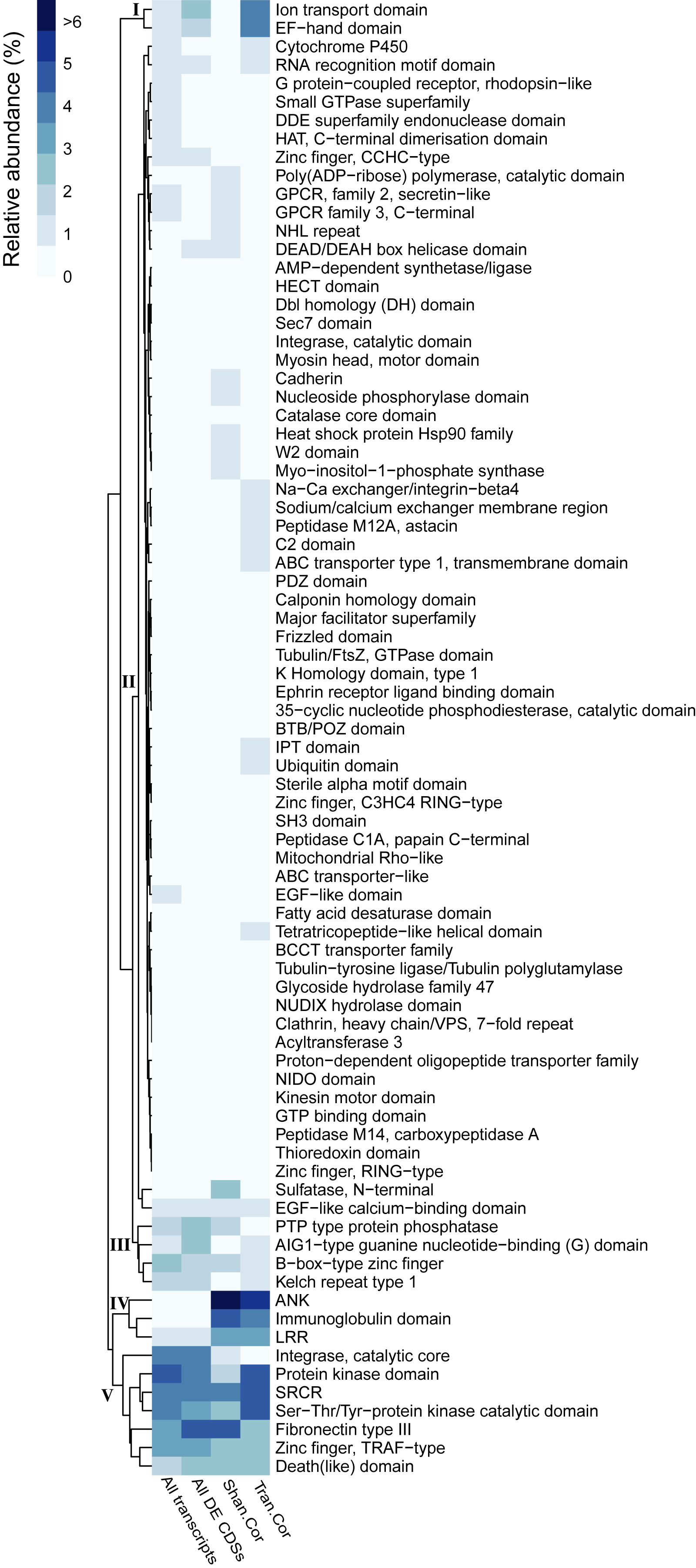


**Fig. S6.** The top 50 most abundant protein families in all transcripts correlated with symbiont functional gene diversity (Shan. Cor.), and DE CDSs correlated with symbiont transcriptional activities (Tran. Cor.).


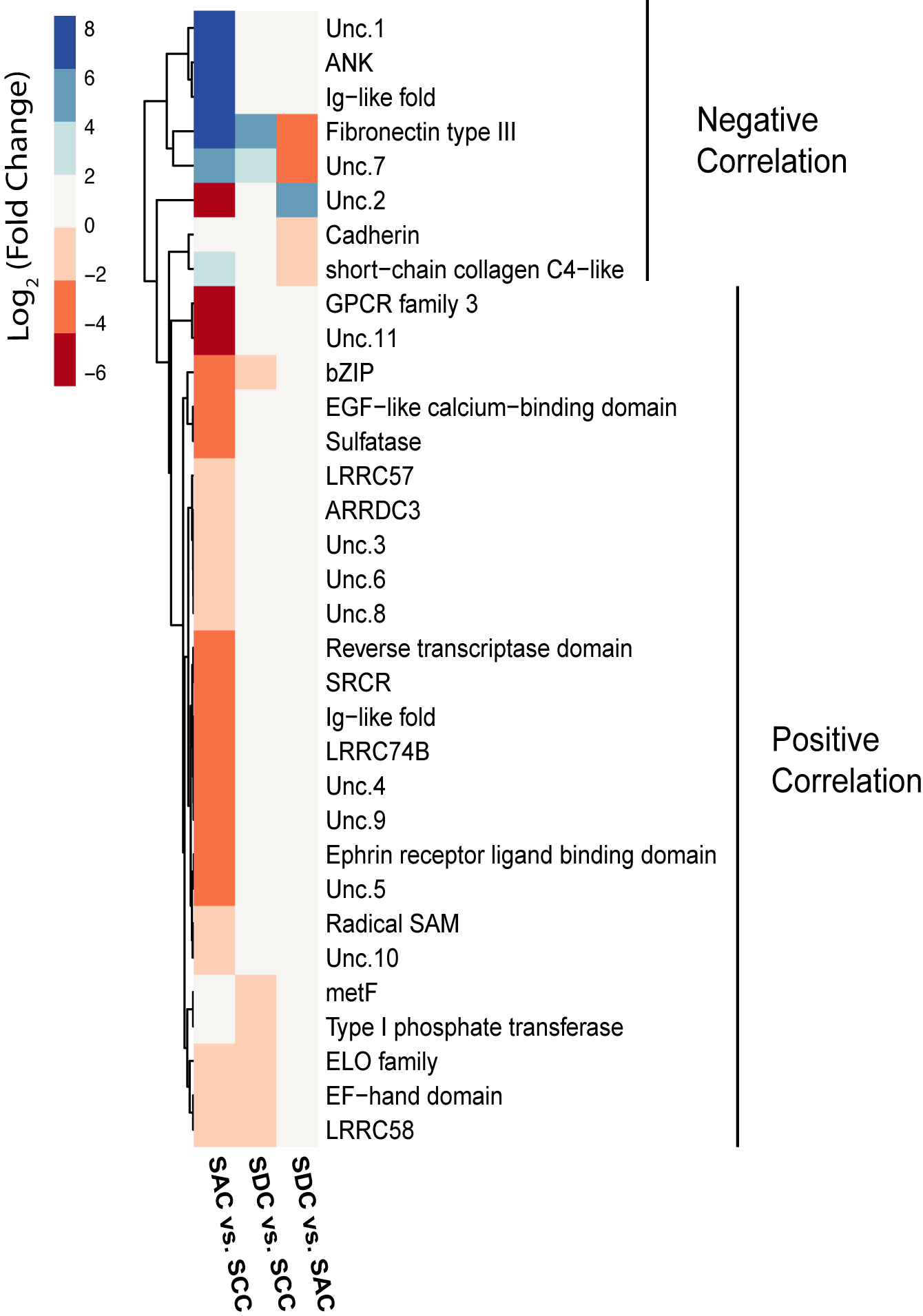


**Fig. S7.** Annotation and foldchange of sponge host’s DE CDSs correlated with both symbiont functional gene diversity and transcriptional activities. Note: SAC –*M. grandis* under alga *G. salicornia*; SCC - *M. grandis* on coral *P. compressa*; SDC - *M. grandis* on the rocks. The C after SD, SA, SC means cDNA sample.

**Table S1**. Primers and barcodes used for amplicon sequencing.

| **Primer** | **Primer sequence (5’-3’)** | **Target** | **Annealing temperature** | **Reference** |
| --- | --- | --- | --- | --- |
| S-D-Bact-0341-b-S-17 | CCTACGGGNGGCWGCAG | Bacterial 16S rRNA V3-V4 | 55℃ | 17 |
| S-D-Bact-0785-a-A-21 | GACTACHVGGGTATCTA ATCC |  |  |  |
| LR0R | ACCCGCTGAACTTAAGC | Fungal 28S rRNA D1-D2 | 52℃ | 20 |
| LR3 | GGTCCGTGTTTCAAGAC |  |  |  |
| **Sample** | **Barcodes (16S)** | **Barcodes (28S)** |  | |
| SAC1 | ACTGACT | ACAGTCA |  |  |
| SAC2 | ACAGTCA | AGACTCT |  |  |
| SAC3 | ACTCTGA | ACTCTGA |  |  |
| SCC1 | TGAGACT | TGTGTCA |  |  |
| SCC2 | TGACTGA | TGACTGA |  |  |
| SCC3 | CAACTGT | TGAGACT |  |  |
| SDC1 | CATCAGA | CACTGAT |  |  |
| SDC2 | CACTTGA | CATGTCT |  |  |
| SDC3 | CACAAGT | CACAAGT |  |  |
| SW | CAGATCA | CAGATCA |  |  |

**Note**: Each PCR reaction was carried following the protocol as suggested in the user manual of KOD Dash® (LDP-101 from TOYOBO).

**Table S2**. Overview of 454 sequencing datasets and α-diversity indices.

|  | **rSW** | **rSDC^d^** | **rSCC^d^** | **rSAC^d^** |
| --- | --- | --- | --- | --- |
| **Reads** | 8565 | 11720±1339.15 | 11525±1251.2 | 11430±1705.11 |
| **No. OTU** | 166 | 177.33±59.53 | 169.66±67.57 | 157.33±23.96 |
| **chao1 ^a^** | 197.71 | 235.23±43.75 | 196.14±71.63 | 208.68±45.7 |
| **Shannon ^a^** | 4.92 | 4.24±0.64 | 4±0.72 | 3.84±0.15 |
| **Simpson ^a^** | 0.93 | 0.89±0.04 | 0.87±0.04 | 0.85±0.02 |
|  | **LrSW ^c^** | **LrSAC** | **LrSCC** | **LrSDC** |
| **Reads** | 1765 | 3922±1415 | 5456±371 | 4938±859 |
| **No. OTU** | 23 | 33±2 | 36±1 | 35±2 |
| **chao1 ^b^** | 24 | 34±1 | 38±2 | 36±2 |
| **Shannon ^b^** | 3.67 | 2.06±0.83 | 1.46±0.11 | 0.94±0.58 |
| **Simpson ^b^** | 0.84 | 0.66±0.2 | 0.52±0.04 | 0.32±0.22 |

**Note**: Name code: r – 16S rRNA, Lr - 28S rRNA. SW – seawater, SAC –*M. grandis* under alga *G. salicornia*; SCC - *M. grandis* on coral *P. compressa*; SDC - *M. grandis* on the rocks. The C after SD, SA, SC means cDNA sample. For sponge datasets, values were showed as mean ± SD (n=3).

a. One-way ANOVA: F_chao1_=0.3925, *P*_chao1_=0.6915; F_shannon_=0.3714, *P*_shannon_=0.7046; F_simpson_=0.6205, *P*_simpson_=0.5689.

b. One-way ANOVA: F_chao1_=2.4925, *P*_chao1_=0.1315; F_shannon_=2.716, *P*_shannon_=0.1807; F_simpson_=2.726, *P*_simpson_=0.184.

c. If non-fungal reads were included, the read count was 6901 and 71 OTUs could be observed. Non-fungal reads were only found in the seawater dataset, *e.g.* reads related to metazoa and protists, which could be due to the specificity of primers and limited knowledge of marine fungal diversity.

d. Containing no more than 3 small OTUs that are further identified as Chloroplasts, which were excluded in the lateral beta-diversity analysis and phylogenetic analysis.

**Table S3.** Pairwise comparison based on weighted_unifrac distance matrix.

| object 1 | object 2 | *p* value | *p* value  (Bonferroni corrected) |
| --- | --- | --- | --- |
| rSAC1 | rSDC3 | 0.39 | 1 |
| rSAC2 | rSDC3 | 0.44 | 1 |
| rSAC3 | rSDC3 | 0.27 | 1 |
| rSCC1 | rSDC3 | 0.49 | 1 |
| rSCC2 | rSDC3 | 0.47 | 1 |
| rSCC3 | rSDC3 | 0.52 | 1 |
| rSDC1 | rSDC3 | 0.96 | 1 |
| rSDC2 | rSDC3 | 0.56 | 1 |
| rSAC1 | rSDC2 | 0.6 | 1 |
| rSAC2 | rSDC2 | 0.44 | 1 |
| rSAC3 | rSDC2 | 0.79 | 1 |
| rSCC1 | rSDC2 | 1 | 1 |
| rSCC2 | rSDC2 | 0.95 | 1 |
| rSCC3 | rSDC2 | 0.97 | 1 |
| rSDC1 | rSDC2 | 0.53 | 1 |
| rSAC1 | rSDC1 | 0.27 | 1 |
| rSAC2 | rSDC1 | 0.61 | 1 |
| rSAC3 | rSDC1 | 0.58 | 1 |
| rSCC1 | rSDC1 | 0.86 | 1 |
| rSCC2 | rSDC1 | 0.82 | 1 |
| rSCC3 | rSDC1 | 0.68 | 1 |
| rSAC1 | rSCC3 | 0.39 | 1 |
| rSAC2 | rSCC3 | 0.68 | 1 |
| rSAC3 | rSCC3 | 0.73 | 1 |
| rSCC1 | rSCC3 | 1 | 1 |
| rSCC2 | rSCC3 | 0.94 | 1 |
| rSAC1 | rSCC2 | 0.3 | 1 |
| rSAC2 | rSCC2 | 0.46 | 1 |
| rSAC3 | rSCC2 | 0.47 | 1 |
| rSCC1 | rSCC2 | 0.95 | 1 |
| rSAC1 | rSCC1 | 0.82 | 1 |
| rSAC2 | rSCC1 | 0.33 | 1 |
| rSAC3 | rSCC1 | 0.8 | 1 |
| rSAC1 | rSAC3 | 0.5 | 1 |
| rSAC2 | rSAC3 | 0.91 | 1 |
| rSAC1 | rSAC2 | 0.13 | 1 |

**Note**: SAC –*M. grandis* under alga *G. salicornia*; SCC - *M. grandis* on coral *P. compressa*; SDC - *M. grandis* on the rocks. The C after SD, SA, SC means cDNA sample.
